# Our Medical Recruits

**Published:** 1874-01

**Authors:** 


Our Medical Recruits—"Tramp, tramp, tramp, the boys are marching"—marching in solid ranks, elbow to elbow, and foot pressed close to foot! Who are they? Whence came they? Where are they going? Boys of every grade of talent, bouyant, happy, marching on to disappointments and hardships innumerable! Who are they? Two full regiments of smiling, happy boys, jolly over the present, hopeful of the future. Whence came they? Two full regiments—jostling and pushing, joking and carousing. Where are they going? Students of medicine, filling College lecture-rooms, on the drill—tramp, tramp, tramp—waiting the word of command, in fighting trim, to march. Soon, diplomas in hand, they will go—tramp, tramp, tramp, on "well fought fields," crowding, pressing those who have gone before, filling every nook and corner; every square inch of unoccupied soil.

Tramp, tramp, tramp, the foot-steps of the regular army resound on the air heavy and sad, bearing hearts full of weariness and woe; tramp, tramp, tramp, the recruits swell their ranks—two thousand every year—overflowing, booming, at flood-tide. Ah! boys—happy boys, tramping gaily; flushed with youth and hopes most bright, you are marching—tramp, tramp, tramp, to many a struggle; to many a hand-to-hand encounter with cupidity, treachery, cunning and avarice—to victory or death. W. T. G.

